# Binding of Androgen- and Estrogen-Like Flavonoids to Their Cognate (Non)Nuclear Receptors: A Comparison by Computational Prediction

**DOI:** 10.3390/molecules26061613

**Published:** 2021-03-14

**Authors:** Giulia D’Arrigo, Eleonora Gianquinto, Giulia Rossetti, Gabriele Cruciani, Stefano Lorenzetti, Francesca Spyrakis

**Affiliations:** 1 Department of Drug Science and Technology, University of Turin, Via Giuria 9, 10125 Turin, Italy; giulia.darrigo@unito.it (G.D.); eleonora.gianquinto@unito.it (E.G.); 2 Institute for Neuroscience and Medicine (INM-9) and Institute for Advanced Simulations (IAS-5) “Computational Biomedicine”, Forschungszentrum Jülich, 52425 Jülich, Germany; 3 Jülich Supercomputing Center (JSC), Forschungszentrum Jülich, 52425 Jülich, Germany; 4 Department of Neurology, RWTH, Aachen University, 52074 Aachen, Germany; g.rossetti@fz-juelich.de; 5 Department of Chemistry, Biology and Biotechnology, University of Perugia, 06123 Perugia, Italy; gabri@chemiome.chm.unipg.it; 6 Istituto Superiore di Sanità (ISS), Department of Food Safety, Nutrition and Veterinary Public Health, Viale Regina Elena 299, 00161 Rome, Italy

**Keywords:** molecular docking, androgens, estrogens, flavonoids, nuclear receptors, G protein-coupled receptors, genomic action, non-genomic action

## Abstract

Flavonoids are plant bioactives that are recognized as hormone-like polyphenols because of their similarity to the endogenous sex steroids 17β-estradiol and testosterone, and to their estrogen- and androgen-like activity. Most efforts to verify flavonoid binding to nuclear receptors (NRs) and explain their action have been focused on ERα, while less attention has been paid to other nuclear and non-nuclear membrane androgen and estrogen receptors. Here, we investigate six flavonoids (apigenin, genistein, luteolin, naringenin, quercetin, and resveratrol) that are widely present in fruits and vegetables, and often used as replacement therapy in menopause. We performed comparative computational docking simulations to predict their capability of binding nuclear receptors ERα, ERβ, ERRβ, ERRγ, androgen receptor (AR), and its variant AR^T877A^ and membrane receptors for androgens, i.e., ZIP9, GPRC6A, OXER1, TRPM8, and estrogens, i.e., G Protein-Coupled Estrogen Receptor (GPER). In agreement with data reported in literature, our results suggest that these flavonoids show a relevant degree of complementarity with both estrogen and androgen NR binding sites, likely triggering genomic-mediated effects. It is noteworthy that reliable protein–ligand complexes and estimated interaction energies were also obtained for some suggested estrogen and androgen membrane receptors, indicating that flavonoids could also exert non-genomic actions. Further investigations are needed to clarify flavonoid multiple genomic and non-genomic effects. Caution in their administration could be necessary, until the safe assumption of these natural molecules that are largely present in food is assured.

## 1. Introduction

Androgens and estrogens are sex steroid hormones typically having two modes of action: a transcriptionally-mediated genomic action and a non-genomic one. The first one is based on the interaction with a specific NR, a dual function protein that is able to translocate within the nucleus, where it acts as a transcription factor. The second occurs through the recognition of plasma membrane specific proteins, acting as extra-nuclear or non-nuclear steroid mediators [1]. Overall, the interaction of androgens and estrogens with their cognate receptors has been demonstrated by either direct binding or indirectly by the activation of downstream effectors of the androgen- or estrogen-regulated signaling pathways [2,3].

### 1.1. Receptors Mediating the Androgenic and Estrogenic Effects 

Molecular mediators of the androgenic action are a NR known as androgen receptor (AR), and different plasma membrane receptors, including the zinc transporter member 9 (ZIP9), the G protein-coupled receptor family C group 6 member A (GPRC6A), the oxoeicosanoid receptor 1 (OXER1), and the calcium channel transient receptor potential melastatin 8 (TRPM8) [4]. Molecular mediators of the estrogenic action include different NRs, such as the estrogen receptors alpha and beta (ERα and ERβ), the estrogen-related receptors alpha, beta and gamma (ERRα, ERRβ and ERRγ), and a G Protein-Coupled Estrogen Receptor (GPER), which is also known as GPR30. Hereafter, we provide a brief description of the role of the mentioned membrane receptors and on their supposed interaction with sex steroids.

ZIP9 belongs to the SLC39A family, whose 14 members regulate zinc transport within the cytoplasm from outside the cell and from intracellular stores. However, little was known regarding the specific physiological role of ZIP9 until 2014, when Thomas and co-workers discovered that ZIP9 is highly expressed in breast and ovarian cancer cell lines and that is bound by androgen hormones [5]. ZIP9 proteins, apart from being involved in zinc transport, can directly activate and/or inhibit G proteins coupled to them. In fact, they can play the double role of zinc transporter and membrane AR (mAR)-activating second messengers [6,7,8]. Both of the actions are mediated by G proteins and they are involved in the androgen-dependent apoptotic response. Dihydrotestosterone (DHT) and testosterone (T) were both demonstrated to bind ZIP9, and bicalutamide was found to inhibit T effects, apparently binding in the same pocket [9].

GPRC6A belongs to class C group 6 subtype A and it is involved in several physiological and pathological activities as bone metabolism, insulin secretion, inflammatory responses, male fertility, prostate tumorigenesis, androgen production in prostate cancer cells, and others [4,10,11]. Several papers suggested that GPRC6A is able to bind different ligands, besides calcium and amino acids, like sex steroid hormones, osteocalcin, and others [12,13], while other works raised doubts on this lack of specificity [14,15]. Apart from this controversy, there are several evidences of T binding to GPRC6A, and computational studies suggested the potential key residues that are involved in T binding [14]. What is not known is the specificity of T binding that could designate this GPCR as another mAR.

OXER1 is another GPCR, whose natural substrates are 5-lipoxygenase metabolites of arachidonic acid, 5-oxoeicosatetraenoic acid (5-oxo-ETE), and 5-eicosatetraenoic acid derivatives [4,16,17,18]. Among the many actions of OXER1, the most relevant are the stimulation of steroidogenesis, cell proliferation and survival of prostate cancer cells, and inflammatory response [19,20]. Hence, OXER1 could be a good target for inflammatory diseases, as well as for prostate cancer, since it is involved in the inhibition of prostate cancer cell apoptosis [19]. In particular, T binding seems to facilitate cell migration and cAMP production [21]. Because T is not the only modulator of OXER1-mediated cAMP levels, additional studies are necessary to clarify T effect on signal transduction and second messenger signaling, and to better investigate androgen binding and its cross-talk with lipid signaling on the same target. Modelling studies have been already performed in this perspective. In particular, the natural substrate 5-oxo-ETE and T were docked in the receptor channel, finding that the two ligands occupy a similar position close to helices 3, 4, and 5 [21]. Stepniewski et al. used molecular modelling simulations to explore the binding of 5-oxo-ETE and derivatives [22], based on the previous information that was provided by Blattermann et al. [23].

TRPM8 is a Ca^2+^-selective cation channel, which is sensitive to menthol, icilin, and the physical stimulus of cold, and is regulated by androgen hormones. TRPM8 is expressed in different tissues such as the male urogenital tract, the intestinal and hepatic tissues, the peripheral nervous system, as well as in cancer tissues [24,25,26]. However, its role in prostate cancer is ambiguous. In fact, TRPM8 is upregulated in the early tumor stage and significantly reduced in advanced androgen-independent stages [26,27], but it has no effect on cell migration, proliferation, and invasion [28,29]. What is certain is its strong binding to T and DHT, which affects its Ca^2+^ channeling activity [30]. According to several experiments, TRPM8 has been recognized as an androgen receptor [31] because of its steroid specificity and androgen binding affinity [32].

As mentioned, the only estrogen membrane receptor known so far is GPER, a seven transmembrane G-protein coupled receptor, which is involved in the modulation of signaling processes that promote tumor growth both in vitro and in vivo [33,34,35]. Its expression is correlated to increased tumor size, distant metastasis, tumor recurrence [36,37], and expression of pro-metastatic genes in ER-negative breast tumors [38]. All of these properties make it a promising therapeutic target for treating different types of tumors [39]. GPER is a specific receptor for the ERs endogenous ligand 17β-estradiol (E2), with a binding affinity of 3–6 nM [40,41], quite lower with respect to the affinity that E2 shows towards ERs (about 0.1–1.0 nM [42]). Other molecules having an agonist/antagonist action towards ERs are known to bind GPER, like, for instance, tamoxifen [43], raloxifene [44], bisphenol A [45], and also plant derived flavonoids, such as genistein [46,47], quercetin [48], and resveratrol [49]. There is, notably, an important structural heterogeneity among molecules that are able to target GPER, clearly hampering the prediction and identification of new ligands [50].

### 1.2. Flavonoids in Androgenic and Estrogenic Signalling Pathways

Several non-steroidal plant bioactives, namely polyphenols and, in particular, flavonoids, recognize the above-mentioned hormone receptors. Although such flavonoids have a lower binding affinity for each single receptor, they can interact with one or more of them, potentially exhibiting an additive binding effect. Such additivity may be representative of a real exposure scenario, in which the dietary intake provides dozens to hundred flavonoids and their metabolites. Historically, several flavonoids have been considered as dietary phytoestrogens [51,52,53] or endocrine disruptors, due to either their direct binding to ERα and ERβ or/and their ability to activate or inactivate an estrogen-like response in vitro in human breast cancer cell lines [3,54]. Additionally, a few in vitro studies that were performed on human-derived prostate cancer cell lines suggested that flavonoids can also target the androgen-dependent signaling pathway by AR activation [55,56].

The first study pointing out the preferential AR activation by estrogens and dietary phytoestrogens was performed using E2 and DHT, and the flavonoids genistein (GEN, a soybean isoflavone) and quercetin (QRC, an ubiquitous flavonol) in a prostate LNCaP cell line with point mutated AR^T877A^ [55]. In this study, Maggiolini and colleagues transformed the LNCaP cells introducing either an ER-binder-dependent gene reporter or an AR/AR^T877A^-binder-dependent gene reporter. As a result, E2, DHT, GEN, and QRC activated the AR- and AR^T877A^-binder-dependent gene reporters, but not the ER-binder-dependent one. Furthermore, all four tested chemicals were able to induce the nuclear translocation of AR.

Among others, in a second study [56], which was performed in LNCaP cells but without using gene reporters, the endogenous sex steroids E2 and DHT were compared to six different flavonoids, GEN, QRC, apigenin, and luteolin (API and LUT, two flavones present in many vegetables and herbal spices), naringenin (NRG, a citrus lemon fruits flavanone), and resveratrol (RESV, a red berry and grape stilbene). The authors characterized the intracellular distribution of both sex steroids and the six flavonoids and showed that only a modest amount of each chemical was located in the nucleus upon each individual treatment. Notably, the nuclear localization of the flavonoids was mostly resembling that of DHT and not of E2, with the exception of QRC. Furthermore, most of the chemicals were located in the microsomal fraction (constituted by plasma membrane and any other intra-cytoplasmic membrane), which suggests their potential recruitment in non-genomic effects, besides the well-known genomic one. All six flavonoids showed a weak, but interesting, anti-androgenic activity, as witnessed by the inhibition of DHT-induced PSA secretion in a non-monotonic dose-response manner [56].

When considering the unclear data regarding the role of flavonoid-type polyphenols as estrogen-like or androgen-like molecules, we performed in silico simulations, to investigate the possible binding of the above-mentioned molecules to the currently known estrogen and androgen nuclear and non-nuclear receptors, in comparison to E2, DHT, and T. To our knowledge, such comprehensive simulations have not been reported so far in the literature. The results underline the plethora of effects that these molecules could exert, thus suggesting caution in their administration and the necessity of performing more accurate studies. 

## 2. Results

**Figure 1:**
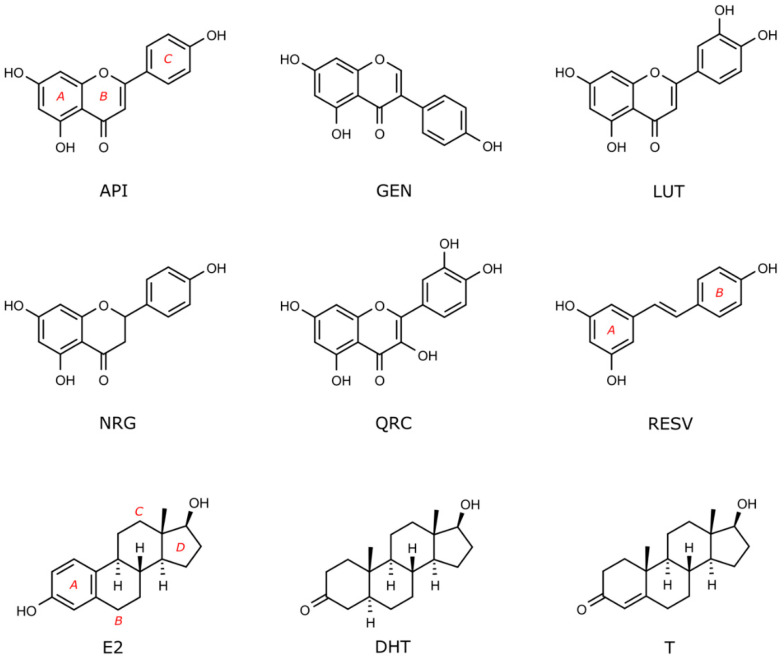
Flavonoids and sex steroid hormones docked in nuclear and membrane receptors shown in Figure 2. Compounds are indicated by their acronyms, as in the introduction and in the following legend: apigenin (API), genistein (GEN), luteolin (LUT), naringenin (NRG), quercetin, (QRC), resveratrol (RESV), 17β-estradiol (E2), dihydrotestosterone (DHT), and testosterone (T).

**Figure 2:**
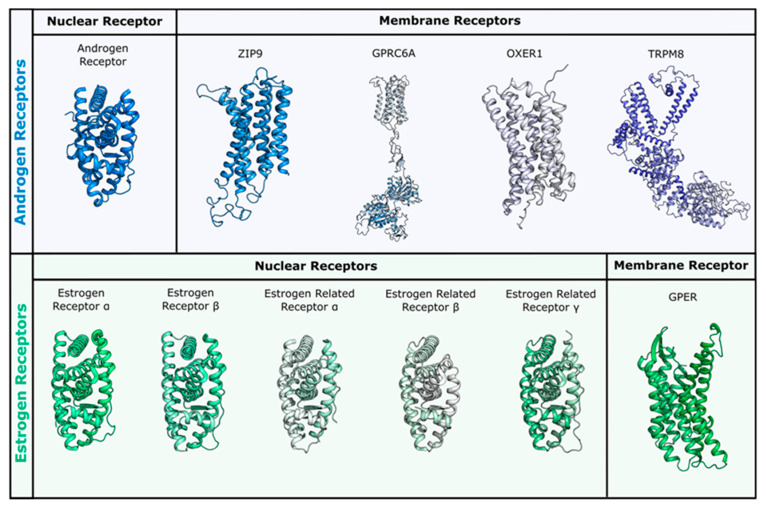
Receptors used for docking studies in this work. Nuclear and membrane receptors for androgen and estrogen sex steroids are reported in panel blue (upper) and green (lower), respectively. All membrane receptors were obtained through homology modelling, while for nuclear receptors the following PDBs were used: androgen receptor (AR) (PDB ID 3l3x for wild type and 3ll for T877A mutant), ERα (PDB ID 3uud) and ERβ (PDB ID 3oll), ERRα (PDB ID 3d24), ERRβ (PDB ID 6lit), and ERRγ (PDB ID 2e2r).

With the aim of investigating the interaction of a series of flavonoids (Figure 1) with nuclear and membrane ARs and ERs, we performed docking studies on the receptors that are reported in Figure 2. In more detail, we considered AR in the wild type and mutated form (AR^T877A^ [57]), and the corresponding membrane receptors that are known to be targeted by T: ZIP9, GPRC6A, OXER1, and TRPM8 [4,58,59,60]. Among ERs, we considered the nuclear ERα, ERβ, ERRα, ERRβ, and ERRγ, and the membrane receptor GPER [39,61]. The sex steroids E2, DHT, and T were also docked in the mentioned receptors, as control and comparison, being ER and AR endogenous compounds, respectively.

Because no three-dimensional structural model is currently available for these membrane receptors, we have built all of them by means of homology modelling techniques. The generated models could represent a valuable reference for computational chemists working in the field and are made available here in the Supporting Information.

### 2.1. Simulations in Nuclear Receptors

Docking studies were first performed in nuclear receptors. The results are reported in Table 1 and in the following paragraphs, and they are separated between ARs and ERs.

#### 2.1.1. Flavonoids and Steroids in Wild Type and Mutated AR

When docked in wild type AR (PDB ID 3l3x), the best scores were obtained by E2, T, and DHT (see Table 1). The docked pose of DHT was superposable to that of the co-crystallized DHT, thus validating the methodology. E2 was scored as equal to the natural AR substrates, consistently with literature data [62] and with our experiments (AlphaLISA™ (Perkin Elmer) assays, data not shown).

**Figure 3:**
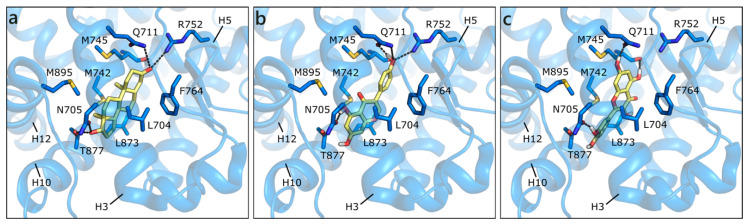
Crystallographic pose of DHT (**a**), and docking poses of GEN (**b**) and QRC (**c**) in wild type AR (PDB ID 3l3x [63]). The protein is shown in transparent blue cartoon, the residues lining the binding site, and the ligands are represented in capped sticks and colored blue and yellow, respectively. Helices and residues lining the pocket are labelled, hydrogen bonds are displayed as black dashed lines.

All of the flavonoids were scored less favorable than steroids, but still in the medium/high nanomolar range, and most of them showed reasonable poses in the binding site (Figure 3). For instance, GEN H-bonds to Asn705, Gln711, and Arg752, as well as DHT, but loses contact with Thr877. Similarly to DHT, hydrophobic interactions are formed with Leu704, Met742, Met745, Phe764, Leu873, and Met895, even if to a minor extent (Figure 3a,b). QRC and LUT were scored a little worse and, indeed, their orientation is inverted and some contacts are lost. Figure 3c shows the docking pose of QRC.

**Figure 4:**
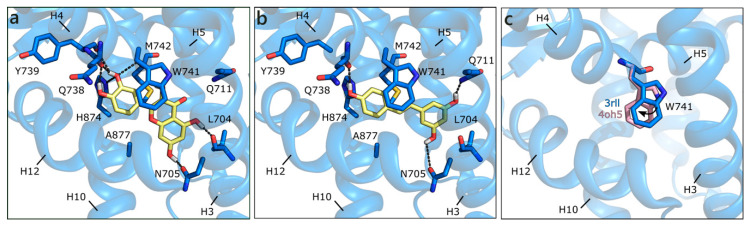
Docking poses of QRC (**a**), and RESV (**b**) in AR^T877A^ (PDB ID 3rll [57]). (**c**) Different orientations of Trp741. The protein is shown in transparent blue cartoon, the residues lining the binding site, and the ligands are shown in capped sticks and colored blue and yellow, respectively. Helices and residues lining the pocket are labelled, hydrogen bonds are displayed as black dashed lines.

The structure used for docking in the AR^T877A^ mutant was 3rll [57], cocrystallized with a bulky B-ring antiandrogen, which induced a different orientation of Gln711 and of Trp741, hanging above the active site. With respect to the structure of rat AR^T877A^ co-crystallized with DHT (PDB ID 1i38 [64]), the resolution is higher and the orientation of Asn705 not flipped. Regardless of the absence of Thr877, which, in the wild type H-bonds the hydroxyl group in ring D, DHT keeps the same orientation (both in the crystal and in the docking model), but the predicted binding energy is less favorable than for the wild type (Table 1). Thanks to the peculiar orientation of Trp741, all of the flavonoids assume a different position in the binding site, establishing H-bonds with Leu704, Asn705, Gln711, Gln738, Tyr739, Met743, and His874. Hydrophobic interactions are formed with Met743 and Met895, and a π-π contact with the same Trp741 is clearly visible. QRC and LUT, having two hydroxyls in ring C, show the most stable network of contacts and present the best score values, being slightly higher than those of DHT, T, and E2 (Figure 4a). Conversely, the other flavonoids still maintain the same orientation, but present less contacts and less favorable scores (Figure 4b). This alternative orientation brings ligands closer to helix12 (H12), which suggests that a slight conformational adjustment of the same helix may occur. As reported in literature [65,66], this might be associated to a partial agonist or antagonist character of these molecules, which could destabilize H12 position, interfering with coactivator recruiting. We also performed induced fit docking simulations to confirm the reliability of this alternative binding pose, obtaining the same results above described.

As mentioned, the alternative pose reported here could be induced by the absence of Thr877, which contributes to orient compounds in the classical steroid-like orientation, but also to the flipping of Trp741. When the same docking procedure was applied using the 4oh5 structure [67], in which Trp741 has a slightly different position (Figure 4c), all of the flavonoids presented the mentioned steroid-like orientation. Thus, compounds can assume different poses, according to the orientation of this key residue that, when moving towards H3, allows for ligands to extend towards the upper region of the binding site (H4). This observation is consistent with crystallographic evidences and underlines the capability of the binding site to adjust to the bound ligand, with a localized induced fit effect.

**Figure 5:**
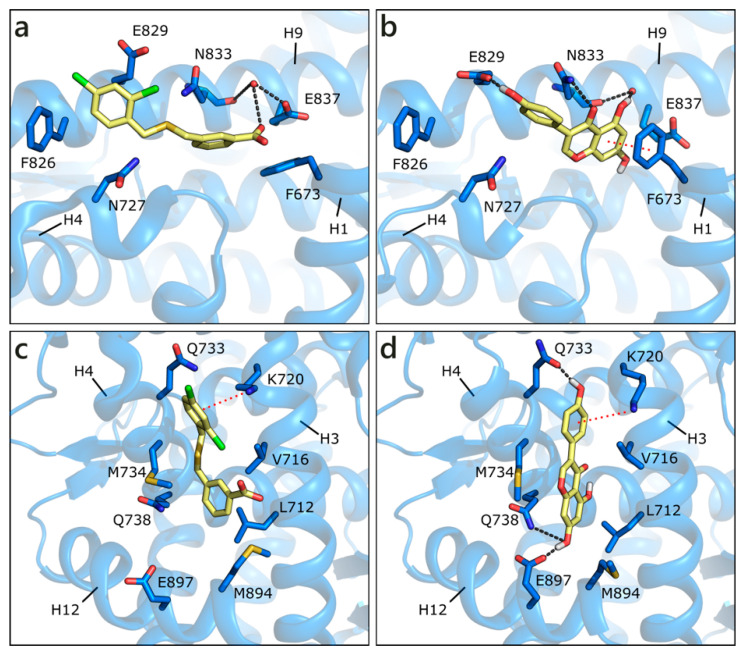
Crystallographic pose of the co-crystallized ligand (**a**), and docking pose of GEN (**b**) in the BF3 pocket of wild type AR (PDB ID 2ylp [68]). Crystallographic pose of the same co-crystallized ligand (**c**), and docking pose of GEN (**d**) in the AF2 pocket of wild type AR (PDB ID 2ylp [68]). The protein is shown in transparent blue cartoon, the residues lining the binding site and the ligands are shown in capped sticks and colored blue and yellow, respectively. Helices and residues lining the pocket are labelled, hydrogen bonds are displayed as black dashed lines. Water molecules are represented as red spheres.

Apart from the orthosteric site, alternative pockets have been investigated. This choice was guided by the presence in the Protein Data Bank of co-crystals, in which small molecules occupy other pockets than the active site. In detail, in PDB ID 2ylp [68], a small ligand (ZINC ID 03445992) occupies the binding function 3 (BF3) site (Figure 5a). This molecule was found through large-scale in silico screening with the aim of finding alternative sites that are less prone to the development of drug resistance, and its inhibition of AR transcriptional activity was proven in cell-based testing. The ligand, possibly protonated on the carboxylic group, interacts with the side chain of Glu837 or, alternatively, with a water molecule also interacting with Asn833 backbone. An additional H-bond is formed with the side chain of Asn833 and hydrophobic interactions with Phe826. GEN obtained the best score, being even higher than the docked co-crystallized ligand, H-bonding to both Glu837, and the mentioned water, Glu829, and Asn833 side chain. A π-π contact with Phe673 is also visible. LUT gains a contact with Asn727, but loses the π-π interaction with Phe673, and presents, overall, a less conserved pose (Figure 5b). Similarly, the other compounds make a lower number of polar and hydrophobic contacts and show more variable poses. These points explain the less favorable scores that were obtained with respect to the active site poses; however, flavonoid interaction with the BF3 pocket cannot be ruled out.

Small molecules have also been found in the activation function 2 (AF2) pocket, which represents the natural binding site of AR co-activators. The binding of a ligand to this pocket could inhibit the co-activator recruitment and, consequently, AR transcriptional activity [69,70,71]. For instance, the same molecule co-crystallized in the BF3 pocket (ZINC ID 03445992) was also found in the AF2 site, in the same crystallographic structure (PDB ID 2ylp [68]). The ligand forms a π-cation contact with Lys720 and hydrophobic interactions with Leu712, Val716, Met734, and Met894 (Figure 5c). An induced fit approach was adopted to position the six flavonoids, while considering that external residues generally present a high level of flexibility. GEN obtained the best score, interacting with Lys720, Gln733, Gln738, and maintaining hydrophobic contacts with Val716 and Met734 (Figure 5d). Similar orientations are assumed by the other flavonoids, which, however, present more variability in the generated docking poses.

#### 2.1.2. Flavonoids and Sex Steroids Hormones in ERα and ERβ, and in ERRα, ERRβ, and ERRγ

The molecules that are reported in Figure 1 were then docked in nuclear ERs, in particular in ERα and ERβ, having E2 as natural substrate, and in orphans ERRα, ERRβ, and ERRγ.

**Figure 6:**
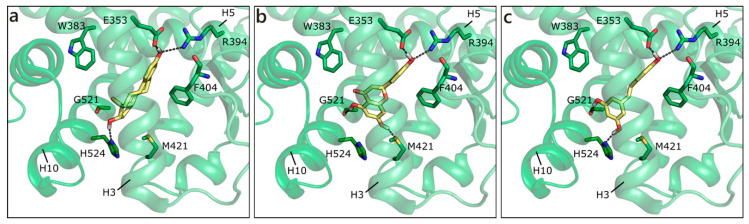
Crystallographic pose of E2 (**a**), and docking poses of NRG (**b**) and RESV (**c**) in ERα (PDB ID 3uud [72]). The protein is shown in transparent green cartoon, the residues lining the binding site and the ligands are shown in capped sticks and colored green and yellow, respectively. Helices and residues lining the pocket are labelled, hydrogen bonds are displayed as black dashed lines.

In ERα, Ε2 obtained the most favorable score, with a docking pose totally resembling that of the co-crystallized ligand (PDB ID 3uud [72]), as shown in Figure 6a. Androgen binding to estrogens receptors occurs with less specificity. So far, only the sex steroid precursor dehydroepiandrosterone (DHEA) and the hormone intermediate androstenediol have been shown to bind ERs with high affinity [73], in particular showing a role via ERβ binding [74]. Androgen binding to ERα has been shown either on laboratory mutated variants [75] or in lower evolutionary species (e.g., trout), although at low affinity [76].

DHT and T assumed the same position of E2, interacting with His524 on one side and Arg394 on the other, only losing the contact with Glu353. They were outscored by NRG and RESV, H-bonding to Glu353, Arg394, Gly521, and Met421 or, alternatively, His524 (Figure 6b,c). Interestingly, our docking studies orient GEN in the same position that is occupied by GEN when co-crystallized with ERα in different PDB entries (PDB IDs 1x7r [77] and 2qa8 [78]), further confirming the reliability of the simulations. Additionally, the docking pose of RESV resembles one of the two possible orientations that were adopted by RESV in complex with ERα (PDB ID 4pp6 [79]). Overall, all the flavonoids maintained the same steroid-like orientation, with a variable number of H-bonds and hydrophobic interactions. Indeed, the capability of flavonoids to act as phytoestrogens is well known and widely reported in the literature [3,53].

A similar trend was maintained in ERβ, where Ε2 obtained the best score value and presented the orientation of the co-crystallized E2 in both ERα and ERβ. The flavonoids were scored in a different order, but with values that support their capability of proficiently interact with the receptor. A small variation in ERβ with respect to ERα can be attributed to the substitutions of Leu384 with Met336, Met421, with Ile373 in the binding site, or for the substitution of more distant residues, resulting in slightly different long-range interactions. Here, GEN, LUT, and QRC obtained the most favorable scores, while NRG, API, and RESV were scored more similar to T (Table 1). Again, the docking pose predicted by our calculations for GEN is consistent with the one that was experimentally observed in the PDB ID 1x7j [77].

**Figure 7:**
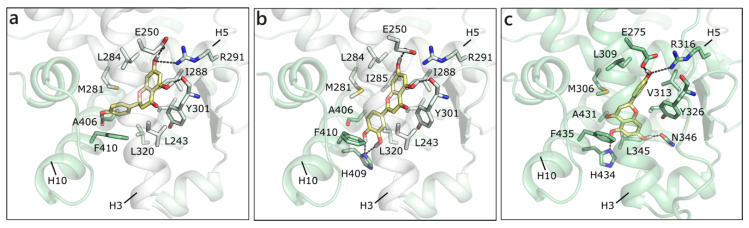
Docking poses of NRG (**a**) and of LUT (**b**) in ERRβ (PDB ID 6lit [84]) and of NRG (**c**) in ERRγ. The protein is shown in transparent light green cartoon, the residues lining the binding site and the ligands are shown in capped sticks and colored light green and yellow, respectively. Helices and residues lining the pocket are labelled, hydrogen bonds are displayed as black dashed lines.

As mentioned, in the list of nuclear estrogen receptors, also ERRα, ERRβ, and ERRγ were considered [80]. The docking in ERRα did not provide any good result, because of a too small binding site almost completely occupied by the side chains of the lining residues (see for instance PDB ID 3d24 [81]). It should be remembered that ERRs do not have any recognized substrate and that ERRα is constitutively active, i.e., it does not require the presence of a ligand to activate the transcription machinery [82,83]. Differently, ERRβ provided interesting results, but an induced fit modality had to be adopted in order to allow active site residues a certain flexibility (PDB ID 6lit [84]). NRG was the best scored, forming polar contacts with Glu250, Arg291, Tyr301, and Ala406, hydrophobic interactions with Phe410, Leu320, Leu243, Met281, Leu284, Ile288, and Ile324, and a π-π contact with Phe410 (Figure 7a). API maintained exactly the same contacts and, indeed, received a very similar score (Table 1). LUT lost the interaction with Ala406, but gained that with His409, and lost the horizontal π-π contact with Phe410 (Figure 7b). QRC, RESV, and GEN maintained the same orientation, but lost some contacts, thus receiving slightly less favorable scores. NRG also received the best score in ERRγ (PDB ID 2e2r [85]) and formed H-bonds with many residues corresponding to those already contacted in ERRβ, i.e., Glu275, Arg316, His203, and Asn346 (Figure 7c). The exact same orientation was maintained by QRC, RESV, and LUT, while many of the poses that were obtained for GEN and API were flipped. 

Overall, these simulations support that the investigated flavonoids, or at least some of them, such as NRG and QRC, might interact with both ERRβ and ERRγ [86,87,88].

### 2.2. Simulations in Membrane Receptors

We also performed docking studies in androgen and estrogen membrane receptors to investigate whether flavonoids could also exert non-genomic actions. In particular, we considered ZIP9, GPRC6A, OXER1, and TRPM8 as T and DHT targets, and GPR30 for E2. Because none of them has been crystallized up to now, we built homology models for all of them (more details are reported in the Materials and Methods section and models are available in SI). The absence of an experimental three-dimensional (3D) structure might bring inaccuracies in structure-based simulations; thus, the following results have to be intended as an indication of binding and not as specific ranking or binding free energy indications.

#### 2.2.1. Flavonoids and Sex Steroids in ZIP9 

**Figure 8:**
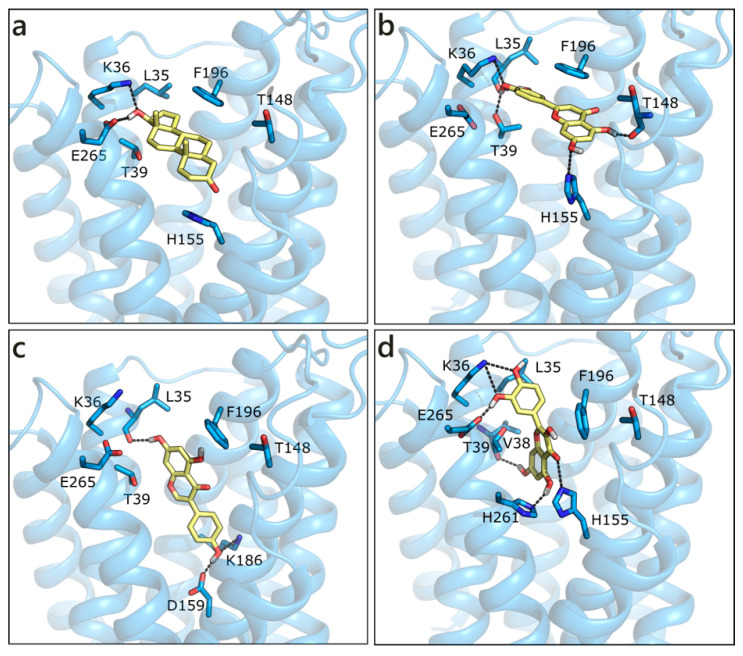
Docking poses of T (**a**), LUT (**b**), GEN (**c**), and QRC (**d**) in the ZIP9 model. The protein is shown in transparent light blue cartoon, the residues lining the binding site and the ligands are shown in capped sticks and colored light blue and yellow, respectively. Helices and residues lining the pocket are labelled, hydrogen bonds are displayed as black dashed lines.

The homology model of ZIP9 was built using, as a template, the structure of a ZIP zinc transporter from *Bordetella bronchiseptica* (PDB ID 5tsa [89]). Despite the poor sequence similarity, the model we obtained presents a good Ramachandran plot (Figure S1). All of the molecules were docked in the central binding cavity of the channel, in the middle of the eight helices. Interestingly, T and DHT, which are known binders of ZIP9, received the less favorable scores (Table 1). Their docked pose is very similar and both ligands form a double H-bond with Lys36 and Glu265 through the hydroxyl group on ring D, while the carbonyl on ring A moiety possibly contacts His155 (Figure 8a). LUT, which is the best scored molecule, presents a higher number of polar contacts with respect to T, involving Leu35, Lys36, Thr39 on one side and Thr148 and His155 on the other, while hydrophobic interactions are made with Thr39 and Phe196 (Figure 8b). GEN, NRG, and QRC have very similar scores. While the pose of NRG resembles that of LUT, GEN and QRC present an orthogonal orientation, as depicted in Figure 8c,d. RESV and API were scored slightly worse and they maintained T orientation.

#### 2.2.2. Flavonoids and Sex Steroids in GPRC6A

**Figure 9:**
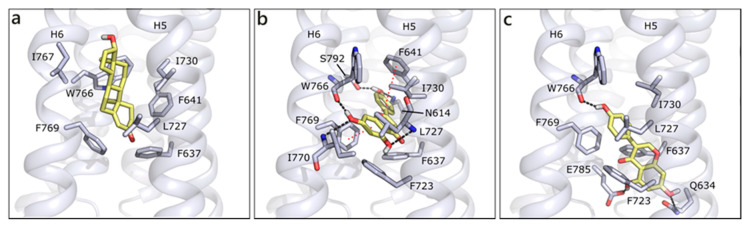
Docking poses of T (**a**), API (**b**), and GEN (**c**) in GPRC6A model. The protein is shown in grey cartoon, the residues lining the binding site and the ligands are shown in capped sticks and colored grey and yellow, respectively. Helices and residues lining the pocket are labelled, hydrogen bonds are displayed as black dashed lines.

We modelled GPRC6A using, as template, the structure of CXCR4 chemokine receptor (PDB ID 6n52 [90]; Figure S2). GPRC6A is formed by a 7-helix transmembrane (7TM) domain and a Venus flytrap (VFT) domain, likely targeted by allosteric effectors as calcium or amino acids [14,91]. We performed docking studies in the 7TM domain, on the basis of the indications that were provided by Pi et al. [14]. According to our molecular docking, T is located between helices 5 and 6 and it forms hydrophobic contacts with Phe637, Phe641, Leu727, Ile730, Trp766, and Phe769, while no polar contact seems to be present. Indeed, the 7TM channel is almost completely lined by hydrophobic residues, which could justify its lack of specificity and capability of binding different ligands. Thanks to their planar conformations, flavonoids are able to be better accommodated in the protein, occupying a more central region and occasionally forming polar interactions. This capability is associated to better scores for flavonoids than for T and DHT, even if poses are quite variable and generally not conserved, likely because of the absence of stable polar contacts. For instance, API, the best scored molecule, H-bonds to Asn614, Ser792 and the backbone of Trp766, Ile770, and Leu727. Hydrophobic interactions are made with Phe637, Phe723, Ile730, and π-π interactions could be formed with Phe769 and Phe641 (Figure 9a). QRC assumes a similar pose, while GEN enters more deeply in the channel, H-bonds to Trp766 backbone on one side and to Gln634 and Glu785 on the other. Additionally, several hydrophobic interactions are formed with the surrounding residues (Figure 9b).

#### 2.2.3. Flavonoids and Sex Steroids in OXER1 

**Figure 10:**
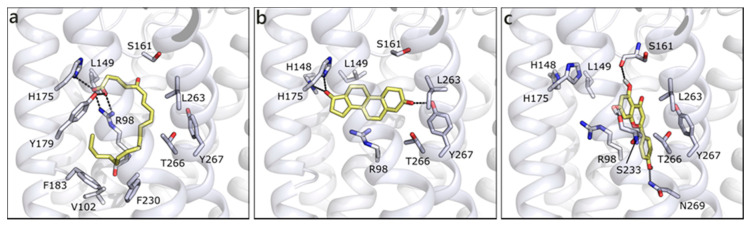
Docking poses of 5-oxo-ETE (**a**), T (**b**), and API (**c**) in OXER1 model. The protein is shown in white cartoon, the residues lining the binding site and the ligands are shown in capped sticks and colored white and yellow, respectively. Helices and residues lining the pocket are labelled, hydrogen bonds are displayed as black dashed lines.

We built the homology model for OXER1 using the structure of the human P2Y purinoreceptor 1 (PDB ID 4xnw) as template. As previously indicated by others [22], the docked pose of 5-oxo-ETE interacts with the key residue Arg98 and with Tyr162, through the carboxylic moiety. The rest of the molecule, mostly hydrophobic, forms hydrophobic contacts with Leu94, Phe183, Phe230, Ile234, Leu263, and Ty266 (Figure 10a). T is scored less favorable (Table 1) than 5-oxo-ETE, H-bonds to His148, His175, Tyr267, and makes hydrophobic interactions with some of the previously mentioned residues (Figure 10b). The best scored molecule of the entire set is API, which presents a quite conserved conformation in the channel and it establishes polar contacts with Ser161 and Ser233 on one side and Asn97 and Asn269 on the other (Figure 10c). Even if the interacting residues are different, the occupied area of the channel is very similar, indeed, a π-cation interaction can be formed with Arg98. All of the other flavonoids maintain the same orientation and interactions, further confirming the reliability of the pose and of their possible engagement with OXER1.

#### 2.2.4. Flavonoids and Sex Steroids in TRPM8

**Figure 11:**
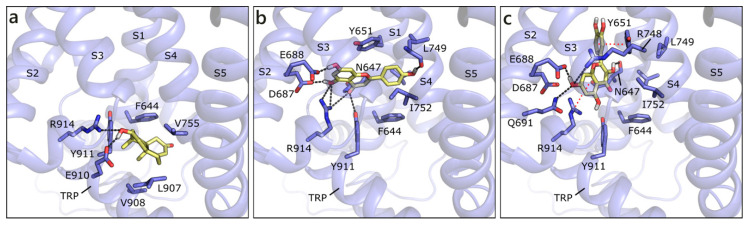
Docking poses of T (**a**), API (**b**), and QRC (**c**) in TRPM8 model. The protein is shown in transparent purple cartoon, the residues lining the binding site and the ligands are shown in capped sticks and colored purple and yellow, respectively. Helices and residues lining the pocket are labelled, hydrogen bonds are displayed as black dashed lines.

We built the human model of TRPM8 on the cryo-EM structure of its homolog from *Ficedula albicollis* in complex with a menthol analogue (PDB ID 6nr2 [92]). The site to dock androgens and flavonoids has been defined, according to the position of the menthol analog in 6nr2 and of the antagonist present in the cryo-EM structure 6o72 from *Parus major* [93], as the transmembrane segment 4 (S4) in the voltage-sensor like domain (VSLD). T and DHT are equally scored and they assume the same position, in which they H-bond to Glu910 and Arg914 and form hydrophobic contacts with Phe644, Val755, and Leu907 (Figure 11a). The best scored flavonoid is API that, as the others, locates slightly over T, and H-bonds to several residues, such as Asn647, Glu688, Leu749, and Arg91. A π-π contact with Tyr651 and hydrophobic interactions with Val648, Phe644, Leu749, and Ile752 are formed (Figure 11b). NRG assumes a similar orientation to API, while QRC, GEN, LUT, and RESV are almost orthogonal to it. For instance, QRC, which also received a good score, forms polar contacts with Asn647 and Glu688, a π-cation contact with Arg914, a π-π one with Tyr651, and hydrophobic interactions with Leu684, Ile752, and Phe919 (Figure 10c). As mentioned, the other flavonoids occupy one of the mentioned positions, but reduce the number or efficacy of interactions. 

#### 2.2.5. Flavonoids and Sex Steroids in GPER

**Figure 12:**
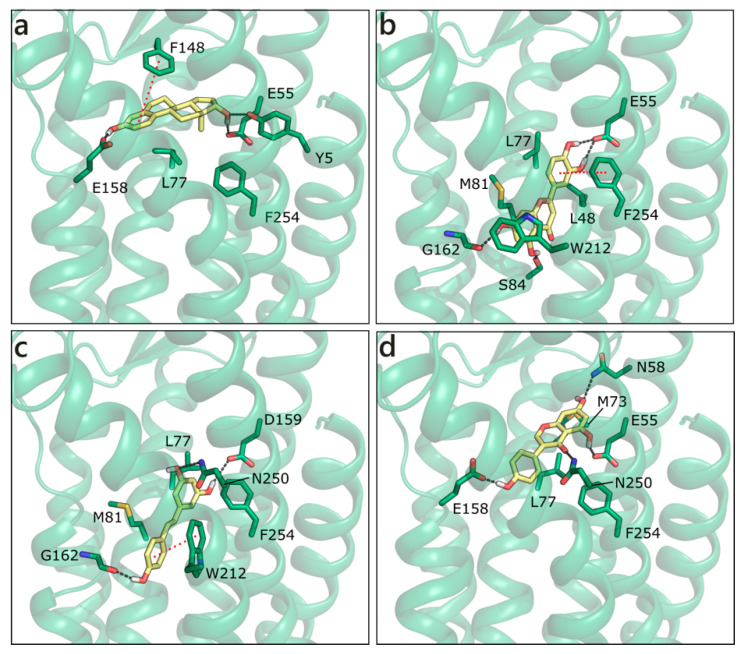
Docking poses of E2 (**a**), LUT (**b**), RESV (**c**), and GEN (**d**) in the G Protein-Coupled Estrogen Receptor (GPER) model. The protein is shown in transparent dark green cartoon, the residues lining the binding site and the ligands are shown in capped sticks and colored dark green and yellow, respectively. Helices and residues lining the pocket are labelled, hydrogen bonds are displayed as black dashed lines.

Homology modelling simulations have been performed to obtain a three-dimensional structure of GPER, while using first as template bovine rhodopsin, then the β2-adrenergic receptor and CXCR4. A number of studies trying to locate the ligand binding site [94,95] and identify new ligands [96,97] has been also published. We have modelled the structure of GPER on the CXCR4 chemokine receptor (PDB ID 3odu [98]; Figure S5), and docked sex steroids and flavonoids in the binding site that was previously defined by others [96,99]. E2 H-bonds to Glu158 with ring A hydroxyl and to Tyr5 and Glu55 with ring D hydroxyl. Moreover, it forms a planar π−π contact with Phe148 and a hydrophobic interaction with Leu77 (Figure 12a). Interestingly, all of the flavonoids apart from GEN are scored more favorably. The best is LUT that forms multiple polar contacts with Glu55, Ser84, Gly162, Asn256, a π−π interaction with Phe254 and hydrophobic interactions with Leu48, Leu77, Met81, and Trp212 (Figure 12b). RESV occupies a very similar position (Figure 12c), contacts Glu55, Gly162, and Asn250, forms a π−π interaction with Trp212 and hydrophobic interactions with the same residues contacted by LUT. NRG and API present very similar scores and contacts, QRC loses some contacts, and GEN assumes a slightly different orientation that allows for the interaction with Asn58, Glu158, Asn250, Phe254, Met73, and Leu77 (Figure 12d).

## 3. Discussion

The six flavonoids analyzed here are all recognized estrogen-like chemicals, which are able to bind estrogen receptors and exert estrogenic or/and anti-estrogenic effects [54,100]. Indeed, phytoestrogens have been described as natural selective ER modulators (SERMs) and, for this reason, they are often used in complementary and alternative therapies to treat menopausal symptoms. They are also sometimes used in women with breast cancer, even if their safety is still not completely proved [101]. Their effects on AR have also been studied [55,56,102,103], while very little is known on their binding to membrane androgen receptors. More information has been reported for flavonoid binding to GPER, as reviewed in Molina et al. [104]. To our knowledge, a comprehensive computational docking analysis, including all estrogen and androgen nuclear and membrane receptors, has not been reported so far. In the light of our findings, hereafter addressed (Section 3.1 and Section 3.2), literature data will be discussed and rationalized (Section 3.3 and Section 3.4).

### 3.1. Flavonoids Binding to Nuclear ERs and ARs

From the performed docking studies, we can infer that the six flavonoids show, as expected, a good complementarity with ERα and ERβ, which confirms their estrogen-like nature, as widely reported in the literature [3,53], and their anti-proliferative character [101,105]. Docking studies in ERRβ and ERRγ also provided very good results, with even better scores than for the docking in ERα and ERβ; in particular, very promising values and poses were obtained for NRG and QRC.

Fang et al. already reported the capability of some phytoestrogens to bind AR, underlining the key role of the two polar groups on rings A and C in anchoring the molecules to the receptor binding site [106]. The analyses carried out here on AR suggested that the investigated flavonoids can bind as well to the wild type and the AR^T877A^ variant, in which they interestingly assume a different orientation. As previously mentioned, this alternative pose could be possibly related to a partial agonist or antagonist character of these molecules, which could alter H12 position, and interfere with coactivator recruiting [65,66]. The presence of two alternative pockets (BF3 and AF2) in AR and AR^T877A^, in which flavonoids could also bind and affect co-activator recruitments, further justify the anti-androgenic activity [56]. However, the level of approximation adopted here does not allow for distinguishing between possible androgenic and non-androgenic effects, which strongly depends on the conformational adjustments that are induced by ligand binding, but also by the pool of co-activator/co-repressors that are present in different tissues.

Our docking simulations showed a slight higher affinity of E2, with respect to DHT and T, towards AR. Accordingly, gene reporter transactivation assays [55] indirectly indicated (i.e., through luciferase activation) a slight higher efficiency of E2 and a slight lower efficiency of GEN and QRC, with respect to DHT, in activating AR-mediated transcription. Interestingly, it was demonstrated that LNCaP cell proliferation that is induced by individual treatments (DHT, E2, GEN, and QRC) was better reversed by the anti-androgenic drug Casodex than by the anti-estrogenic compound ICI 182,780. Even more interestingly, Casodex was more efficient on QRC-, E2-, and GEN-induced proliferation than on the DHT-induced one, whereas ICI 182,780 decreased the cell proliferation induced by each of the four tested chemicals much less. This seems to suggest that cell proliferation could be under an androgenic control, more than an estrogenic control, and further confirms that flavonoids proficiently bind AR.

### 3.2. Flavonoids Binding to Membrane ERs and ARs

Docking studies in membrane receptors also provided quite interesting results, even if ligand poses were less conserved and generally characterized by lower scores (Table 1). This is quite reasonable, when considering the totally different architecture of non-nuclear membrane receptors and of their binding sites, larger and less defined with respect to those of nuclear receptors. Moreover, as previously mentioned, in the absence of 3D experimental structures, all of the membrane receptors were built by means of homology modelling. This brings a certain degree of inaccuracy in the results and the score values should be only considered as an indication of binding. 

Regarding hypothetical mARs, the simulations performed in ZIP9 sustain the hypothesis that flavonoids, as well as T, can bind the receptor. The best results were obtained for LUT, GEN, NRG, and QRC, which occupy the same region of T in the receptor cavity and assumed a similar pose. Good and consistent results were also obtained for TRPM8, for which the ligand poses, the interactions formed, and the corresponding scores strongly suggest that the investigated flavonoids can bind to TRPM8, as androgens do [30,32]. Even if these simulations cannot predict whether flavonoids might have an agonist or antagonist effect on TRPM8, it is interesting to note that TRPM8 blockers could be used in pathologies that are exacerbated by cold as, for instance, asthma, chronic cough, or to reduced cold hypersensitivity from nerve damage [107,108]. Docking simulations performed in OXER1 also returned promising predictions [21], while, in the case of GPRC6, quite variable poses and very few polar interactions were obtained for both T and flavonoids. Indeed, the receptor binding site is almost only lined by hydrophobic residues, which suggests that ligand binding could be not very specific, as reported in the literature [14,15].

The results that were obtained for GPER were quite consistent in terms of poses and in agreement with respect to previous analyses [97,99]. When considering the availability of experimental data confirming binding of some flavonoids to GPER [46,47,48,49], we can reasonably assume that flavonoid effects is not only mediated by the interaction with nuclear receptors, but also with the membrane counterpart.

### 3.3. Sex Steroids and Flavonoids Localization in a Human Prostate Cell Line

The analyses reported here allowed for us to partially rationalize the intracellular distribution of sex steroids and flavonoids previously investigated in LNCaP human prostate cell lines [56]. It has been reported that E2 shows higher nuclear localization than DHT and flavonoids. This peculiarity could be possibly explained by the high complementarity of E2, not only towards ERα and ERβ, but also towards the AR and the AR^T877A^ variant. Moreover, with respect to DHT, E2 seems to bind to a lower number of membrane receptors, i.e., only GPER [39]. It has been also shown that, when LNCaP cells were treated with a combination of one sex steroid (DHT or E2) and one of the six flavonoids here investigated, the effective intracellular concentration of the molecules, and their distribution in the nuclear fraction and the microsomal one (plasma membrane and all other cytoplasmic membranes except for the nuclear one) changed, according to the co-treatment combination. This seems to suggest that sex steroids and flavonoids bind the same targets and affect each other’s localization.

In general, all of the flavonoids seem to be mainly located in the LNCaP microsomal fraction [56]. This is in total agreement with the hypothesized flavonoid binding to membrane receptors and with the experimental evidence of T and E2 binding to the same targets. It is quite reasonable that some flavonoids, as well as sex steroid hormones, distribute in membranes, even if the experimental and estimated affinity for nuclear receptor is higher. Indeed, LUT and RESV, having the highest fraction of microsomal distribution among flavonoids [56], also present scores and poses among the most favorable in membrane receptors. The effect of a few polyphenols on membrane receptors has been recently reported. In particular, epigallocatechin-3-gallate was demonstrated to exert both agonist and antagonist effects on GPRC6A [109], while the lignan sesamin performed as an antagonist for TRPM8 [110].

The presence of membrane receptors in LNCaP cells is supported by the observation that all of these receptors are expressed in human prostate epithelium [12,21,111] and in human-derived cells [112,113,114]. In general, membrane receptors are expressed in the male reproductive tissues both in vivo and in vitro [4,21,115]. 

### 3.4. Flavonoids Anti-Androgenic Effect

In LNCaP cells, E2 and the six flavonoids, when co-administered with DHT, were found to partially inhibit the PSA secretion that is induced by the same DHT [56]. Experiments were run with a time-resolved fluoroimmunoassay on LNCaP cells expressing the AR^T877A^ variant. It has to be noted that E2 by itself induced a lower PSA secretion, thus indirectly confirming our results that E2 perfectly binds AR active site, but decreases the DHT-dependent PSA secretion. This effect again supports the capability of E2 of binding AR and activating the transcription machinery, even if to a less extent than the natural DHT substrate. Flavonoids, with the only exception of LUT at concentration > 10 μΜ, did not induce PSA secretion. Rather, they inhibited the DHT-dependent PSA secretion, thus showing an anti-androgenic action. This effect can occur through binding at the active site in a conformation that does not allow the recruitment of co-activators or through binding at alternative allosteric sites. As reported above, in our docking studies, all six flavonoids assumed an alternative orientation in AR^T877A^ orienting closer to H12, and resembling the pose of known antagonists [65,66]. Indeed, this different pose could induce a slight conformational adjustment of H12 and a consequent difficulty in co-activator recruiting. The binding to allosteric sites preventing co-activator recruitment is an alternative that might explain flavonoid anti-androgenic effects. We have, indeed, reported that the binding to BF3 and AF2 sites is also feasible. The occupation of the AF2 cavity would obviously prevent the interaction with co-activators, but also the BF3 site has proven to inhibit AR transcriptional activity [68].

The binding of flavonoids to AR^T877A^ active site is further supported by the effect they have on the intracellular distribution of DHT, as previously reported by us [56]. For instance, QRC has a relevant localization in the nucleus (≈20%) and, if co-administered with DHT, almost completely reduces the nuclear localization of the latter, thus suggesting a competition at the active site level or binding at an allosteric site. It is interesting to note that QRC was top ranked when docked in the AR^T877A^ active site (Table 1). A similar effect, even if to a less extent, was observed for LUT, the second top-ranked flavonoid in AR^T877A^, and by API (Table 1). RESV basically does not change DHT nuclear concentration, thus suggesting a preferential localization of the flavonoid to alternative binding site rather than the active one.

## 4. Materials and Methods 

### 4.1. Homology Modelling

The homology modelling of the membrane steroid receptors (ZIP9, GPR30, GPRC6A, OXER1, and TRPM8) was performed using the Swiss-Model Protein Modelling Server [116] (https://swissmodel.expasy.org/ access date 25 October 2020). The 3D model of ZIP9 was obtained using the BbZIP structure from *Bordetella bronchiseptica* (PDB ID 5tsa) as a template, sharing a sequence identity of 19,44%. No metal ions were added to the final structure. GPER was modelled using the structure of the CXCR4 chemokine receptor (PDB ID 3odu, sequence identity: 26.26%), while GPRC6A, using the structure of the metabotropic glutamate receptor 5 (PDB ID 6n52, sequence identity: 30.52%). Finally, the structural models of OXER1 and TRPM8 were obtained from the human P2Y purinoreceptor 1 (PDB ID 4xnw, sequence identity: 23.49%) and the TRPM8 homolog from *Ficedula albicollis* (PDB ID 6nr2, sequence identity: 79.89%), respectively.

### 4.2. Molecular Docking

The panel of nine compounds, including steroids (E2, T, DHT) and flavonoids (API, GEN, LUT, NRG, QRC, RESV), were submitted to docking calculations towards the set of 10 receptors (nuclear receptors and membrane nuclear receptors). Prior to docking, protein structures were prepared while using the Protein Preparation Wizard in Maestro and ionized at a pH of 7.5 using PROPKA. When available, the co-crystallized ligands were used as the centroid of the receptor grid. 

When available, the co-crystallized ligands in the NRs X-ray coordinates or in the template structures that were used for homology modelling were taken as reference to define the receptor docking grid. If not, in combination with literature data, a pocket search using the FLAPsite algorithm, as implemented in FLAP, was performed to look for putative binding sites [117,118].

The six flavonoids and E2, T, and DHT were docked in the orthosteric pocket of wild type AR (PDB ID 3L3X), AR^T877A^ (PDB ID 3rll), ERα (PDB ID: 3uud), and ERβ (PDB ID 3oll) using Glide SP (standard precision) [119], generating up to 10 poses for each system. The IFD (Induced-fit docking) [120] was used instead for ERRα (PDB ID 3d24), ERRβ (PDB ID 6lit), ERRγ (PDB ID 2e2r), and the membrane steroid receptors’ models, and to additionally dock the flavonoid compounds in the AF2-pocket and BF3-site of AR (PDB ID 2ylp). The IFD protocol was performed, as follows: an initial softened-potential docking, using a scaling factor of 0.50 kcal/mol for both the ligands and receptors, was performed with Glide to generate up to 10 poses. A subsequent refinement step was applied to all the residues within 5.0 Å of each of the 10 ligand poses using Prime. Finally, the structures within 30 kcal/mol of the minimum energy structure were used for redocking with Glide SP.

## 5. Conclusions

In the last years, polyphenols and flavonoids have been suggested as breast and prostate cancer preventatives [121,122,123] and administered as adjuvants in the treatment of menopause and osteoporosis [124,125]. Because these compounds are present in plants and seeds, they can be easily assumed with the diet, although with a limited bioavailability. Soybean, which is a fundamental supplement of Asian diet, represents an important source of flavonoids, in particular GEN [126], and people in Asia show a lower rate of hormone-dependent cancers, mainly breast and prostate ones [121,127] Indeed, it is known that: (i) GEN can block PSA induction mediated by AR [122,123]; (ii) API, GEN, LUT, NRG, QRC, and RESV can reduce DHT-induced PSA secretion to a different extent in a non-linear manner [56]; (iii) API exerts a protective role against prostate cancer [128]; and, (iv) soy beverage can decrease PSA level in prostate cancer patients [129,130]. These effects appear to be directly related to their capability of binding hormone receptors, as already reported by others [102,106] and as detailed in our simulations. This capability has mainly been justified by their structural similarity to the endogenous E2 and DHT substrates. However, the way in which these molecules exert an agonist or antagonist effect on nuclear receptors is not easily predictable, and it likely depends on the specific co-activator/co-repressor population of different tissues. Even less is known regarding their binding to non-nuclear receptors and the possible consequent effects. 

Here, we have performed a rather exhaustive study on the binding of six flavonoids, i.e., apigenin, genistein, luteolin, naringenin, quercetin, and resveratrol, to estrogen and androgen nuclear and membrane receptors, finding a good complementarity for most of the cases. These results seem to suggest that such plant derived chemicals also interact with non-nuclear estrogen and androgen receptors, exerting non-genomic actions. This hypothesis, which was partially supported by few experimental findings, deserves attention and more investigations. 

## Figures and Tables

**Table 1:** Docking scores for the investigated flavonoids and steroids in nuclear and membrane receptors.

		Nuclear Receptors									Membrane Receptors				
		Androgen				Estrogen					Androgen				Estrogen
		AR^wt^	AR^T877A^	AR BF3 Site	AR AF2 Site	ERα	ERβ	ERRα *	ERRβ	ERRγ	ZIP9	GPRC6A	OXER1	TRPM8	GPER
**Steroids**	**E2**	−11.510	−9.448	-	-	−10.944	−10.242	-	-	-	-	-	-	-	−7.455
	**DHT**	−11.296	−9.053	-	-	−9.692	−9.040	-	-	-	−6.899	−10.688	−7.544	−7.676	-
	**T**	−11.340	−9.865	-	-	−9.897	−8.883	-	-	-	−7.031	−9.942	−9.184	−7.705	-
**Flavonoids**	**API**	−9.778	−9.891	−6.652	−6.453	−9.698	−8.679	-	−11.319	−10.144	−7.579	−11.392	−10.940	−10.534	−8.047
	**GEN**	−10.136	−8.531	−8.104	−8.501	−9.820	−9.853	-	−10.328	−10.341	−8.405	−11.136	−10.159	−7.892	−7.010
	**LUT**	−9.715	−9.910	−6.896	−6.475	−9.311	−9.346	-	−10.996	−10.977	−8.903	−10.120	−8.570	−7.589	−9.080
	**NRG**	−9.723	−9.147	−6.637	−6.479	−10.215	−8.937	-	−11.449	−11.905	−8.222	−10.411	−9.426	−7.898	−8.132
	**QRC**	−9.373	−9.940	−5.860	−6.521	−8.911	−9.255	-	−10.618	−11.192	−8.373	−10.494	−9.562	−8.808	−7.726
	**RESV**	−9.743	−8.248	−6.425	−7.776	−10.098	−8.256	-	−10.515	−11	−7.698	−10.086	−9.353	−6.766	−8.791

* No predicted binding pose.

## Data Availability

Data are available upon request from the corresponding authors.

## Supplementary Materials

The following are available online. Figure S1: Structural architecture of nuclear receptors, Figure S2: Ramachandran plot for ZIP9 model, Figure S3: Ramachandran plot for GPRC6A model, Figure S4: Ramachandran plot for OXER1 model, Figure S5: Ramachandran plot for TRPM8 model, Figure S6: Ramachandran plot for GPER model, file with the generated models for membrane receptors (models.zip).
