# Supplementary material for: Binding of Androgen- and Estrogen-Like Flavonoids to Their Cognate (Non)Nuclear Receptors: A Comparison by Computational Prediction
Source: Molecules. 2021 Mar 14;26(6):1613. doi: 10.3390/molecules26061613 (PMC8001607; doi:10.3390/molecules26061613)
Supplement: Supplementary file 1 [file molecules-26-01613-s001.zip › SI/Supplementary Information.docx]

**Supplementary Information**

Binding of androgen- and estrogen-like flavonoids to their cognate (non)nuclear receptors: a comparison by computational prediction

Giulia D’Arrigo ^1^, Eleonora Gianquinto ^1^, Gabriele Cruciani ^2^, Stefano Lorenzetti ^3,^*, Francesca Spyrakis ^1,^*

^1^ Department of Drug Science and Technology, University of Turin, via Giuria 9, 10125, Turin, Italy, [giulia.darrigo@unito.it](mailto:giulia.darrigo@unito.it) (G.D.); [eleonora.gianquinto@unito.it](mailto:eleonora.gianquinto@unito.it) (E.G.); [francesca.spyrakis@unito.it](mailto:francesca.spyrakis@unito.it) (F.S.)

^2^ Department of Chemistry, Biology and Biotechnology, University of Perugia, 06123, Perugia, Italy; gabri@chemiome.chm.unipg.it

^3^ Istituto Superiore di Sanità (ISS), Department of Food Safety, Nutrition and Veterinary Public Health; [stefano.lorenzetti@iss.it](mailto:stefano.lorenzetti@iss.it)

***** Correspondence: [stefano.lorenzetti@iss.it](mailto:stefano.lorenzetti@iss.it) (S.L.), [francesca.spyrakis@unito.it](mailto:francesca.spyrakis@unito.it) (F.S.)

**Table of content**

**Figure S1. Structural architecture of nuclear receptors……………………………………………..……………..S2**

**Figure S2. Ramachandran plot for ZIP9 model………………………………………………..……………………S3**

**Figure S3. Ramachandran plot for GPRC6A model…….…………………………………………………………S4**

**Figure S4. Ramachandran plot for OXER1 model…………………………….……………………………………S5**

**Figure S5. Ramachandran plot for TRPM8 model…...……………………………………………………………S6**

**Figure S6. Ramachandran plot for GPER model……………………………………………….……….…………S7**

**Figure S1. Structural architecture of nuclear receptors.** Protein folding is shown as cartoons and helices are labelled.

**
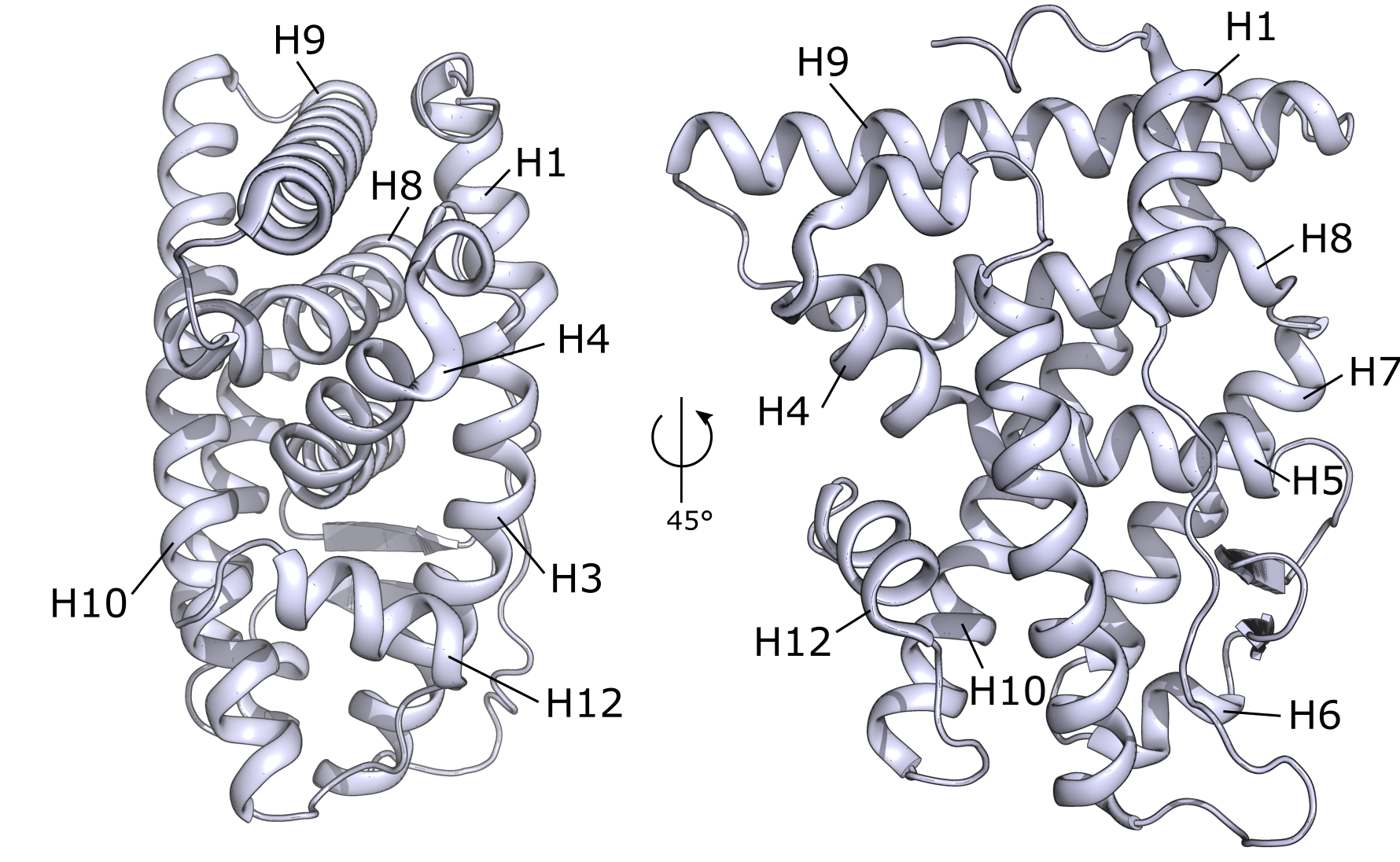
**

**Figure S2. Ramachandran plot for ZIP9 homology model.** The points in the Ramachandran plot are colored according to the QMEAN parameter which gives an estimation of the residue quality. The color scale goes from red to blue indicating a bad or good quality, respectively.

**
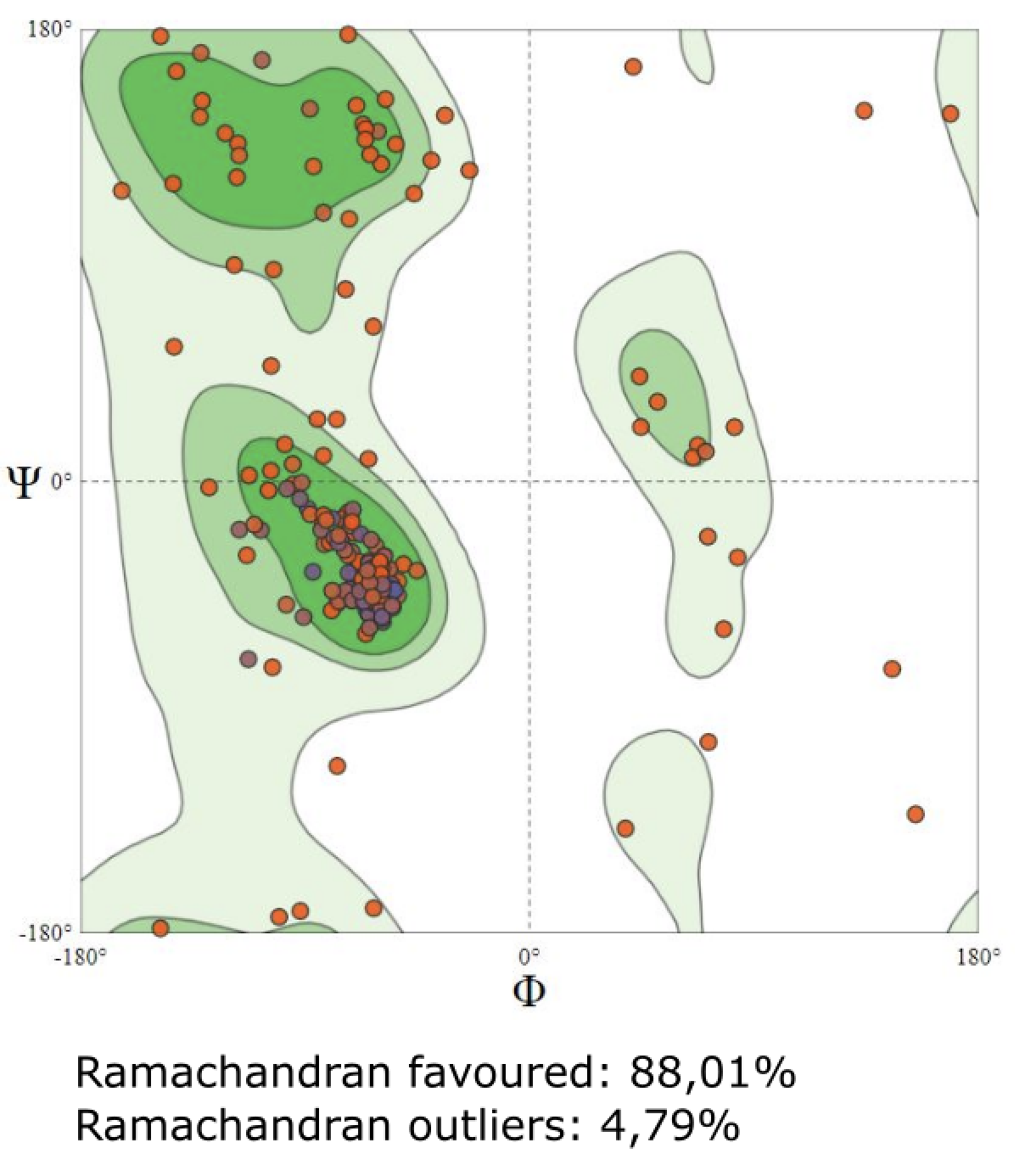
**

**Figure S3. Ramachandran plot for GPRC6A homology model.** The points in the Ramachandran plot are colored according to the QMEAN parameter which gives an estimation of the residue quality. The color scale goes from red to blue indicating a bad or good quality, respectively.

**
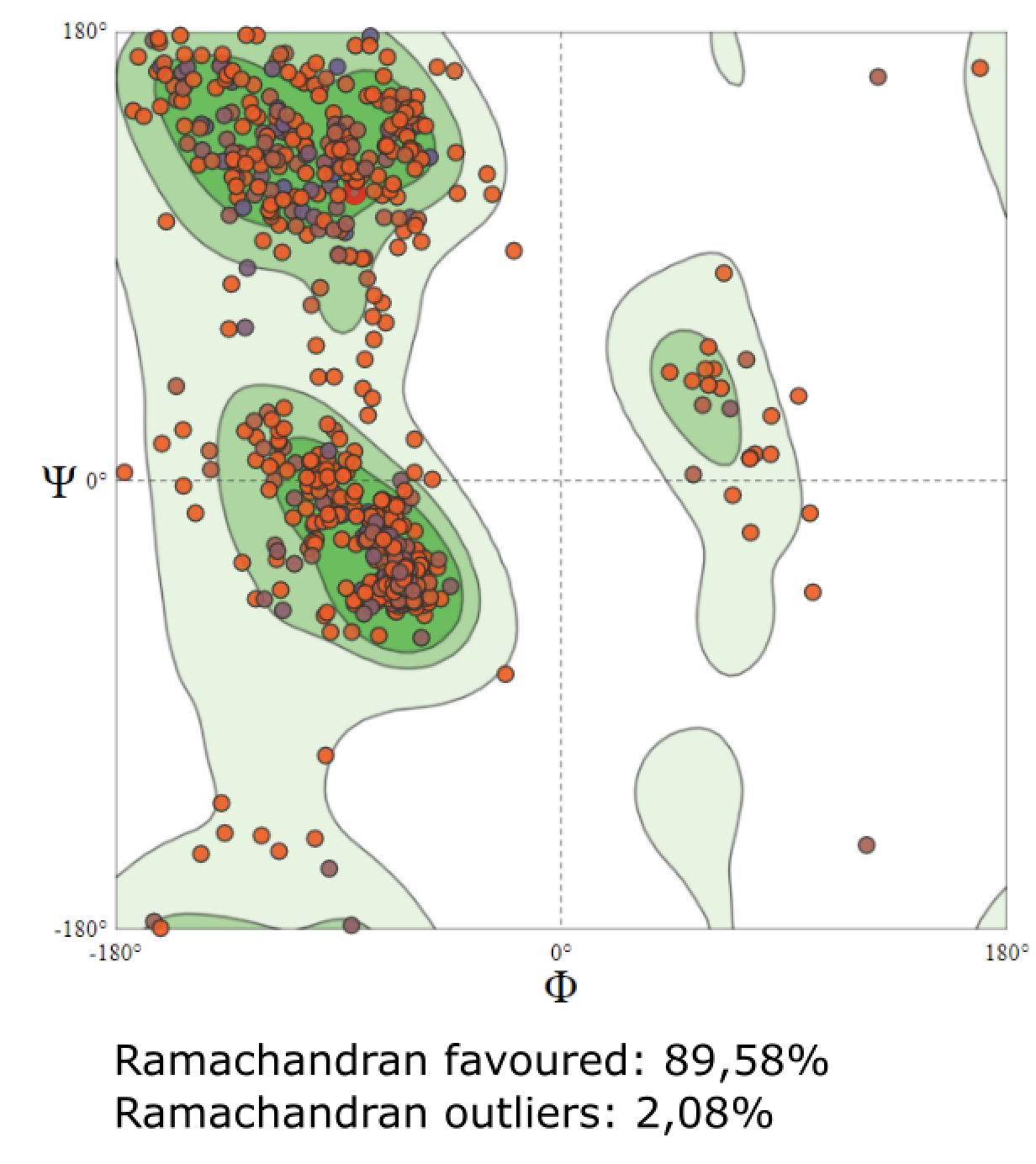
**

**Figure S4. Ramachandran plot for OXER1 homology model.** The points in the Ramachandran plot are colored according to the QMEAN parameter which gives an estimation of the residue quality. The color scale goes from red to blue indicating a bad or good quality, respectively.

**
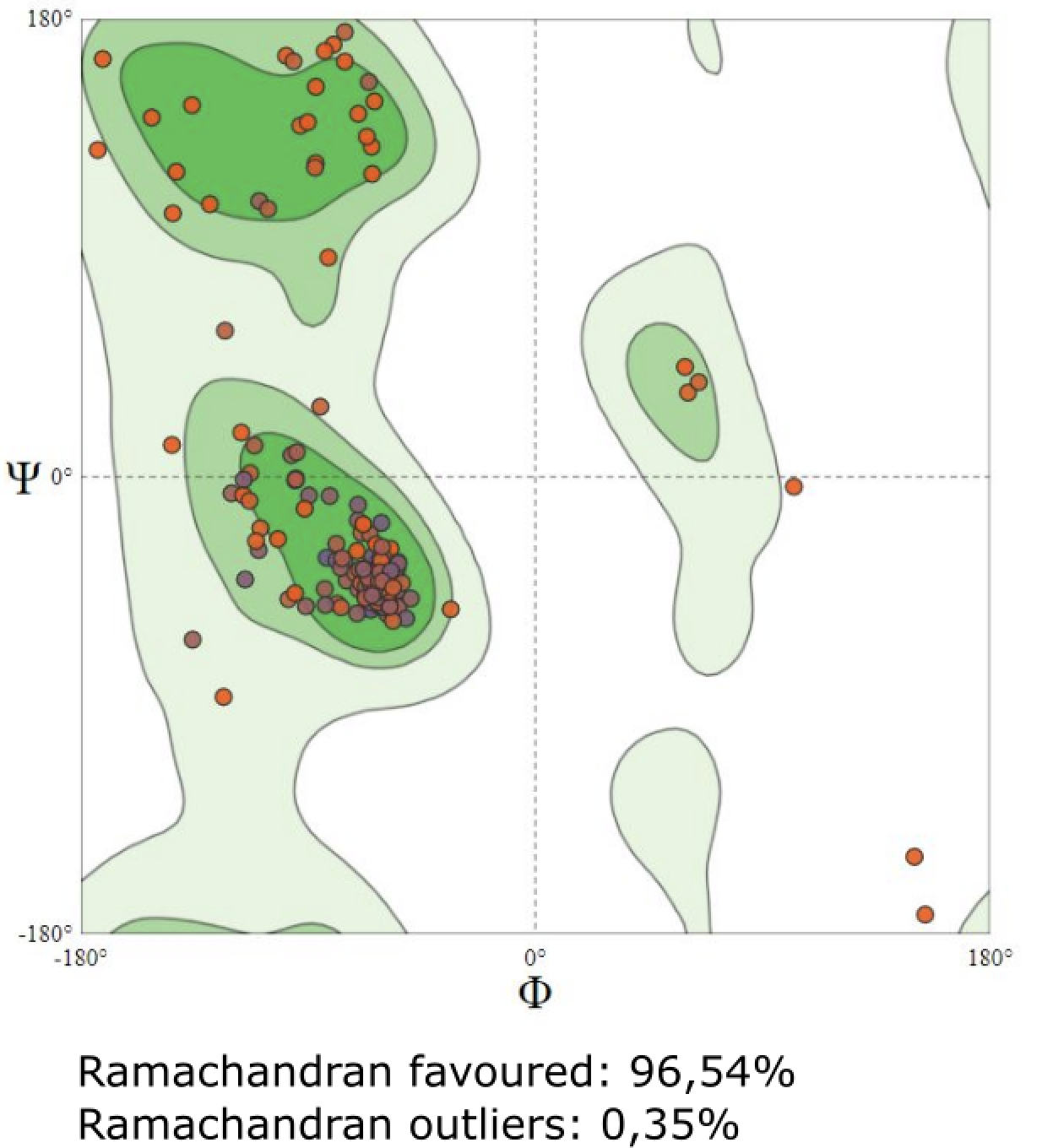
**

**Figure S5. Ramachandran plot for TRPM8 homology model.** The points in the Ramachandran plot are colored according to the QMEAN parameter which gives an estimation of the residue quality. The color scale goes from red to blue indicating a bad or good quality, respectively.

**
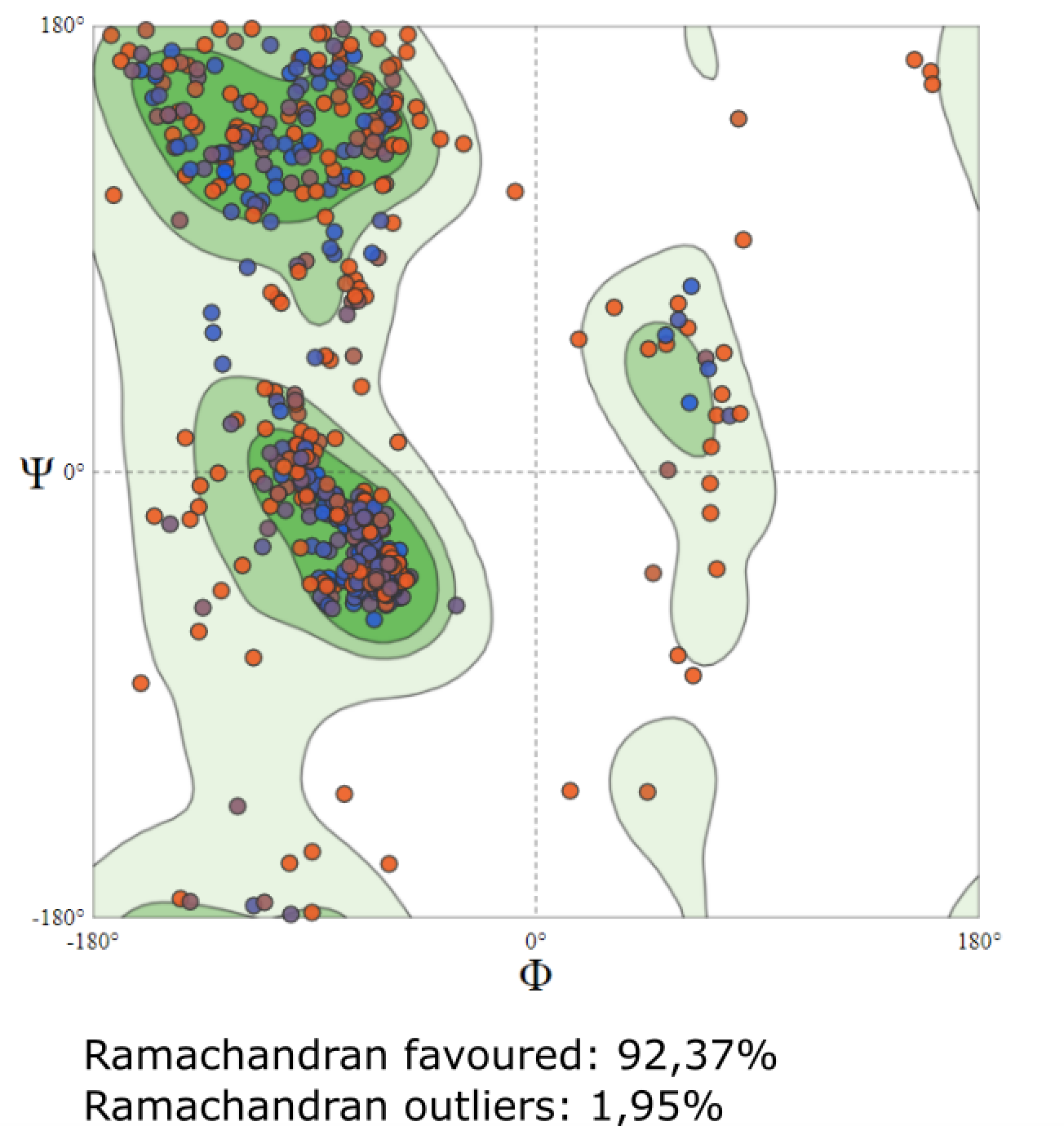
**

**Figure S6. Ramachandran plot for GPR30 homology model.** The points in the Ramachandran plot are colored according to the QMEAN parameter which gives an estimation of the residue quality. The color scale goes from red to blue indicating a bad or good quality, respectively.

**
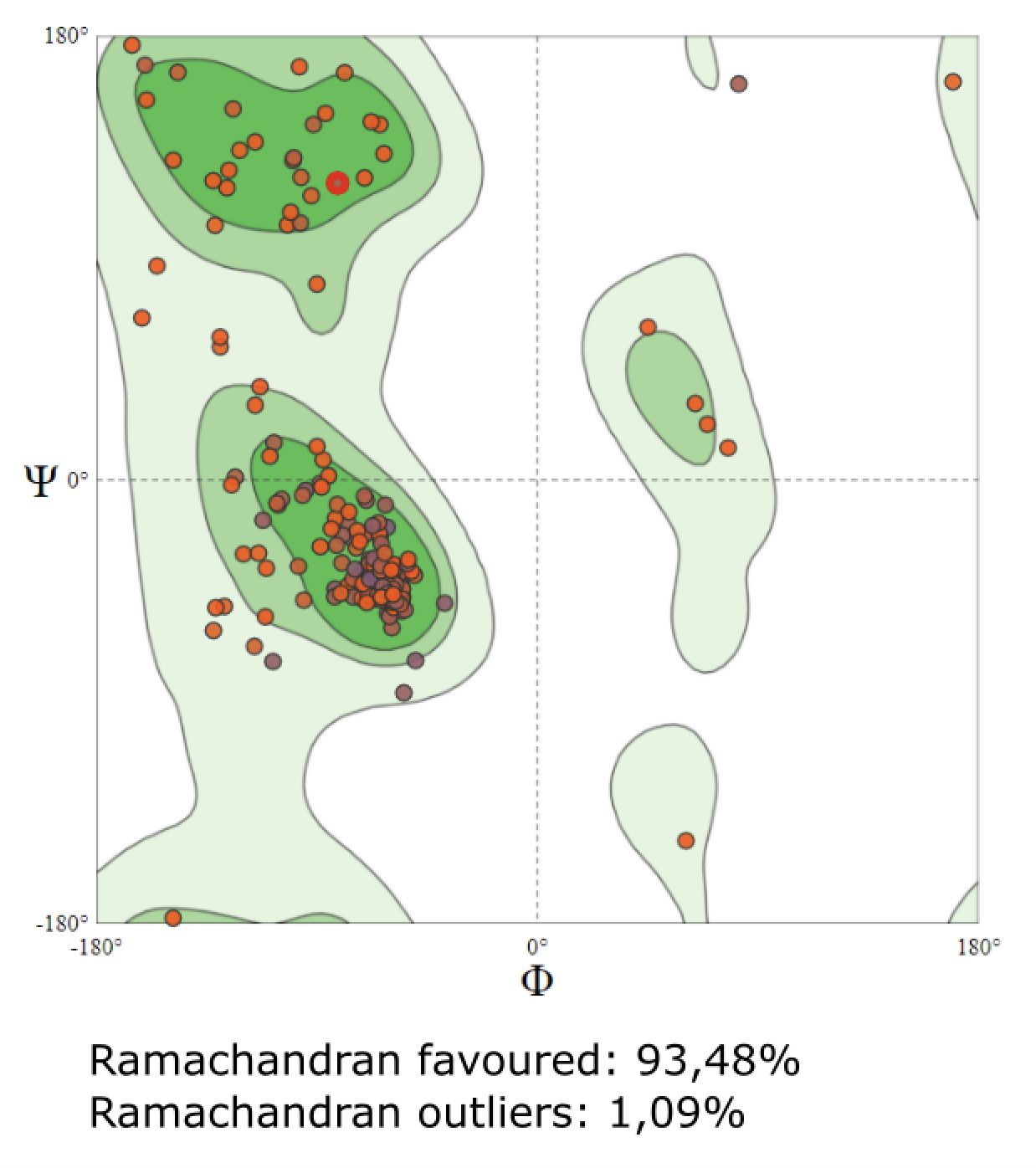
**
